# Investigating the Relationship Between Neuronal Cell Death and Early DNA Methylation After Ischemic Injury

**DOI:** 10.3389/fnins.2020.581915

**Published:** 2020-10-14

**Authors:** Mayumi Asada, Hideki Hayashi, Kenjiro Murakami, Kento Kikuiri, Ryotaro Kaneko, Bo Yuan, Norio Takagi

**Affiliations:** ^1^Department of Applied Biochemistry, Tokyo University of Pharmacy and Life Sciences, Hachioji, Japan; ^2^Laboratory of Pharmacology, Faculty of Pharmacy and Pharmaceutical Sciences, School of Pharmacy, Josai University, Sakado, Japan

**Keywords:** DNA methylation, neuronal cell death, DNMT3a, transient MCAO, NMDA, cerebral ischemia, primary cultured cortical neuron

## Abstract

Cerebral ischemia induces neuronal cell death and causes various kinds of brain dysfunction. Therefore, prevention of neuronal cell death is most essential for protection of the brain. On the other hand, it has been reported that epigenetics including DNA methylation plays a pivotal role in pathogenesis of some diseases such as cancer. Accumulating evidences indicate that aberrant DNA methylation is related to cell death. However, DNA methylation after cerebral ischemia has not been fully understood yet. The aim of this present study was to investigate the relationships between DNA methylation and neuronal cell death after cerebral ischemia. We examined DNA methylation under the ischemic condition by using transient middle cerebral artery occlusion and reperfusion (MCAO/R) model rats and *N*-methyl-D-aspartate (NMDA)–treated cortical neurons in primary culture. In this study, we demonstrated that DNA methylation increased in these neurons 24 h after MCAO/R and that DNA methylation, possibly through activation of DNA methyltransferases (DNMT) 3a, increased in such neurons immediately after NMDA treatment. Furthermore, NMDA-treated neurons were protected by treatment with a DNMT inhibitor that were accompanied by inhibition of DNA methylation. Our results showed that DNA methylation would be an initiation factor of neuronal cell death and that inhibition of such methylation could become an effective therapeutic strategy for stroke.

## Introduction

DNA methylation is one of the types of epigenetic modification, which is the mechanism for regulation of gene expression without changes in the DNA sequence. In embryogenesis, this mechanism induces genome imprinting and X chromosome inactivation, which have important implications for normal development (Csankovszki et al., 2001; Kaneda et al., 2004). The epigenetic mark of DNA methylation occurs at the cytosine of CpG dinucleotides in their gene promoter, and DNA methyltransferases (DNMTs) are known as an epigenetic writer that transfers methyl groups from S-adenosylmethionine to the 5-position of cytosine residues. Three types of DNMTs are known as enzymes that transfer methyl groups to DNA (Moore et al., 2013). DNMT1 plays a key role in the maintenance of methylation, whereas DNMT3a and DNMT3b work as *de novo* DNA methylation enzymes. Accumulating evidences indicate that alterations of DNA methylation are related to the pathogenesis of various diseases, not only cancer (Gopisetty et al., 2006) but also central nervous system diseases (Kang et al., 2013; Lu et al., 2013). Especially, it has been reported that aberrant DNA methylation may be related to neuronal cell death (Chestnut et al., 2011; Farinelli et al., 2014).

Nowadays, the population is aging all over the world, and it is thought that the number of patients of age-related diseases will increase more and more in the future (Amani et al., 2019a). Stroke is one of such diseases and leading causes of death around the world. Thrombolytic therapy using tissue plasminogen activator (t-PA) is known as a promising treatment for ischemic stroke. However, t-PA has narrow therapeutic time window because of its severe hemorrhagic side effects that induce inflammation of brain capillaries and subsequently severe injury to neurons after stroke (Amani et al., 2019b). Therefore, the use of t-PA is restricted to selected patients only, and patients who have not been treated with t-PA will suffer from serious aftereffects. Thus, to develop novel therapeutic strategies for stroke is needed to rescue such patients.

Ischemic stroke leads to neuronal cell death induced by abundant Ca^2+^ influx from the extracellular space through the *N*-methyl-D-aspartate (NMDA) receptor, which is a type of glutamate receptor. This Ca^2+^ influx activates various cell death–related signals that then induce neuronal cell death. In the central nervous system, neuronal cell death leads to brain dysfunction such as motor and cognitive dysfunction. Therefore, preventing neuronal cell death is necessary for the development of therapeutic strategies for stroke. It was reported that inhibition of DNA methylation has the ability to reduce infarct volume in mild focal brain ischemia (Endres et al., 2000). Moreover, another study reported that ischemic preconditioning, which induces tolerance to ischemic cell death, decreases global DNA methylation *in vitro* (Meller et al., 2015). These accumulating evidences indicate that the regulation of DNA methylation can be an effective therapeutic strategy for stroke. However, the details of the relationships between neuronal cell death and DNA methylation after severe ischemic damage are not yet fully understood. Therefore, we investigated changes in the levels of DNMT1, DNMT3a, and DNMT3b, in addition to measuring DNA methylation, in the brain after severe cerebral ischemia and examined the effects of DNMT inhibitors on excitotoxic injury in cultured neuronal cell to define a therapeutic target.

## Materials and Methods

### Surgical Procedures of Transient Focal Cerebral Ischemia

Middle cerebral artery occlusion and reperfusion (MCAO/R), which mimics transient focal cerebral ischemia, was carried out on male Sprague–Dawley rats (7 weeks, weighing between 220 and 260 g; SLC, Shizuoka, Japan) in this study. The rats had free access to food and water and were maintained based on the National Institutes of Health (NIH) Guide for the Care and Use of Laboratory Animals and the Guideline of Experimental Animal Care issued by the Prime Minister’s Office of Japan. The protocol of animal experiments was approved by the Committee of Animal Care and Welfare of Tokyo University of Pharmacy and Life Sciences.

MCAO/R was performed by the method described previously (Yamada et al., 2019). Briefly, anesthesia was induced with 4% isoflurane and followed by maintenance with 2% isoflurane. The right common carotid artery, external carotid artery, and internal carotid artery were first exposed. Then, a 4-0 nylon surgical suture with a silicone-coated tip was inserted from the external carotid artery to the beginning of the right middle cerebral artery. The operated rats were tested for neurological deficits before reperfusion according to the method described previously (Bederson et al., 1986). The rats demonstrating consistent circling toward the contralateral side and a reduced resistance to a lateral push toward the contralateral side were used in the present study. The suture was withdrawn 90 min after the occlusion to allow reperfusion. Those rats that did not undergo any surgical procedure were used as naive animals. Sham-operated rats received exactly the same surgical procedure but without MCAO.

To determine the effect of DNMT inhibitor on infarct area after MCAO, vehicle or RG108, an inhibitor of DNMT, was injected into the right intracerebroventricular over 3 min (posterior; 0.7–0.8 mm from bregma, lateral; 1.5 mm from bregma, depth; 3.5 mm from the skull surface) just before the surgery for MCAO/R. The infarct size was evaluated by 2,3,5-triphenyltetrazolium chloride (TTC)–staining of brain slices. Coronal sections with a 2 mm width were made at 24 h after MCAO/R, and the slices were incubated with 2% TTC in physiological saline. TTC-unstained areas were analyzed by use of an image analyzer (NIH image 1.63, NIH, Bethesda, MD, United States).

### Western Immunoblotting

In the animal study, rats were sacrificed by decapitation 24 h after surgery for cerebral ischemia- or sham-operated rats. Tissue was harvested as slices of 4–6 mm from the olfactory bulb and then separated into ischemic core and penumbra, as previously reported (Yamada et al., 2019). Each part was sonicated in lysis buffer containing 1% TritonX-100, 0.1% deoxycholic acid, and 1 mM EDTA in 50 mM Tris-buffered saline with protease inhibitors (Roche Diagnostics Co., Mannheim, Germany). After measuring the protein concentration, samples were heated at 95°C for 5 min in sample buffer comprising 62.5 mM Tris–HCl (pH 6.8), 10% glycerol, 2% sodium dodecyl sulfate (SDS), and 5% β-mercaptoethanol. The cortical neurons were washed thrice with phosphate-buffered saline (PBS). After that, the cells were harvested in sample buffer and heated in the same way. Western immunoblotting was performed according to standard protocols. Proteins applied onto the gel were separated by SDS–polyacrylamide gel electrophoresis and then transferred to polyvinylidene difluoride membranes at 80 V for 2 h. The following primary antibodies were used: rabbit monoclonal anti-DNMT1 (dilution, 1:1,000; cat. no. 5032, Cell Signaling Technology Inc., Danvers, MA, United States), rabbit monoclonal anti-DNMT3a (dilution, 1:1,000; cat. no. 3598, Cell Signaling Technology Inc.), rabbit polyclonal anti-DNMT3b (dilution, 1:1,000; cat. no. ab2851, Abcam, Cambridge, United Kingdom), and mouse monoclonal anti-β-actin (dilution, 1:10,000; cat. no. A5441, Sigma-Aldrich, St. Louis, MO, United States) antibody. Subsequently, the protein blots were washed and incubated with the appropriate secondary antibodies (dilution, 1:5,000; Pierce Biotechnology, Rockford, IL, United States). Immunoreactive proteins were detected by using ImmunoStar basic (FUJIFILM Wako, Osaka, Japan), ImmunoStar zeta (FUJIFILM Wako), or West Femto (Pierce Biotechnology). Quantification was performed by using computerized densitometry (LuminographII, ATTO Co., Tokyo, Japan) and an image analyzer (CS Analyzer, ATTO Co.).

### Quantitative Reverse Transcription–Polymerase Chain Reaction

The total RNA was extracted from the cortex of rats and cortical neurons in primary culture by using an RNA extraction kit, Isogen II (Nippon Gene, Tokyo, Japan). The concentrations of extracted RNAs were quantified by use of a BioSpec-nano (Shimazu Corp., Kyoto, Japan). Then the cDNAs were synthesized from 500 ng of total RNAs by using ReverTra Ace^®^ qPCR RT Master Mix with gDNA Remover (TOYOBO Co., Ltd., Tokyo, Japan). Quantitative reverse transcription–polymerase chain reaction (PCR) was performed by using THUNDERBIRD^®^ SYBR qPCR Mix (TOYOBO Co., Ltd.) on a CFX Connect Real-Time PCR Detection System (Bio-Rad Laboratories, Hercules, CA, United States). Data were normalized to 18S rRNA mRNA or β-actin mRNA expression and analyzed by the 2-ΔΔCt method. Primers used in the present study were as follow: DNMT1—forward, 5′-AACCACTCAGCATTCCCGTA-3′; reverse, 5′-TGCTGGTAC TTCAGGTCAGG-3′. DNMT3a—forward, 5′-AAGGTCAAGG AGATCATTGATGAAC-3′; reverse, 5′-CATTGAGGCTCCCA CATGAGAT-3′. DNMT3b —forward, 5′-GATGTGACACCTA AGAGCAGCAGTAC-3′; reverse, 5′-CAAACTCCTTGTCATC CTGATACTCA-3′. UHRF1 —forward, 5′-ATGTGGCGGGT ATTCATGGTC-3′; reverse, 5′-GCTGTACGCTTGTTGCCAG AG-3′. 18S rRNA—forward, 5′-CGGACAGGATTGACAGAT TG-3′; reverse, 5′-CAAATCGCTCCACCAACTAA-3′. β-Actin —forward, 5′-TGCTATGTTGCCCTAGACTTCG-3′; reverse, 5′-GTTGGCATAGAGGTCTTTACGG-3′.

### Immunohistochemistry

One, six, sixteen, and twenty four hours after surgery, rats were perfused via the heart with 4% paraformaldehyde (PFA) in 0.1 M phosphate buffer. The brains were quickly removed and fixed with 4% PFA overnight. After that, they were immersed in 10–30% sucrose in 0.1 M phosphate buffer. Next, the brains were cut into 4 mm thick coronal slabs, which were subsequently embedded in Neg50 (Richard-Allan Scientific, Kalamazoo, MI, United States) and cut into 10 μm sections with a cryostat. The sections were then washed thrice for 5 min each time with PBS. Treatment with DNase I for 5-methylcytosine (5mC) immunostaining was performed as previously reported with minor modifications (Tkatchenko, 2006; Zhang et al., 2017). Briefly, the sections were incubated with 100 U/mL DNase I (cat. no. LS002139, Worthington Biochemical Co., Lakewood, NJ, United States) in DNase I buffer comprising 10 mM MgCl_2_ in 50 mM Tris–HCl (pH 7.5) for 30 min at 37°C. Next, they were incubated in 50 mM Tris–HCl (pH 7.5) for 10 min at 70°C for inactivation of the DNase I and then cooled in 50 mM Tris–HCl (pH 7.5) on ice for 3 min. Antigen retrieval was performed by use of HistoVT One (cat. no. 06380, Nacalai tesque Inc., Kyoto, Japan) for 20 min at 70°C. After three washings with PBS, the sections were immersed in PBS containing 0.3% TritonX-100 for 15 min at room temperature to make the membranes permeable to antibody. Blocking was performed with Blocking One Histo (cat. no. 06349-64, Nacalai Tesque) for 1 h at room temperature, after which the primary antibody was applied at 4°C. The next day, the sections were incubated with the appropriate secondary antibody for 1 h at room temperature. The following primary antibodies were used: mouse anti-NeuN (cat. no. ab104224, Abcam), mouse anti-GFAP (cat. no. 556327, BD Biosciences, Franklin Lakes, NJ, United States), and rabbit monoclonal anti-5mC (cat. no. 28692, Cell Signaling Technology Inc.) antibody. The secondary antibodies used were the following: Alexa Fluor 488–labeled goat anti-mouse IgG (cat. no. A11029; Invitrogen, Carlsbad, CA, United States) and Alexa Fluor 594–labeled goat anti-rabbit IgG (cat. no. A11037; Invitrogen). Fluorescence was detected by using an Olympus fluorescence microscope (IX-71; Olympus, Tokyo, Japan). Fluorescent images were loaded into the MetaMorph software program (Molecular Devices, Sunnyvale, CA, United States). Based on the background fluorescence and the size of cells, the antibody-labeled cells of the cerebral cortex were observed by using the MetaMorph software program. The numbers of cells were counted by using the MetaMorph software program in two sections per animal, which corresponded to coronal coordinates of 1.70 mm to -0.30 mm from bregma. Three chosen areas (1.3 mm^2^ each) in the core region and one (1.3 mm^2^) in the penumbra region were examined per section.

### Primary Cultures of Rat Cortical Neurons

Primary cultures were prepared as previously described (Yamada et al., 2019). In brief, cerebral cortices of Sprague–Dawley fetal rats at embryonic day 16 (SLC, Shizuoka, Japan) were dissected and digested with 0.25% trypsin (Invitrogen) in PBS for 20 min at 37°C. After trituration in Neurobasal medium (Invitrogen) containing 10% fetal bovine serum by use of a fire-polished Pasteur pipet, the isolated cortical cells were suspended in Neurobasal medium containing 0.5 mM glutamine, 2% B27 supplement (Invitrogen), and 1% penicillin-streptomycin (FUJIFILM Wako). These cortical cells were plated at a density of 200,000 cells/well in 24-well plates (Falcon, Corning, NY, United States) coated with poly-D-lysine (FUJIFILM Wako) and then cultured for 10 days before experiments. The rats were maintained according to the NIH Guide for the Care and Use of Laboratory Animals and the Guideline for Experimental Animal Care issued by the Prime Minister’s Office of Japan. All experimental procedures were approved by the Committee of Animal Care and Welfare of Tokyo University of Pharmacy and Life Sciences.

### NMDA Treatment

NMDA treatment was performed as previously described (Yamada et al., 2019). In brief, cortical neurons in primary culture at 10 days *in vitro* (DIV) were washed twice for 15 min each time at 37°C with 250 μL/well Hanks balanced salt solution (HBSS; Invitrogen) containing 2.4 mM CaCl_2_ and 20 mM HEPES without magnesium, which can block the NMDA receptor (HBSS buffer). Subsequently, the neurons were incubated with HBSS buffer, containing 30 μM NMDA and 10 μM glycine for 15 min at 37°C. After treatment with or without NMDA, cortical neurons were cultured for the desired times in the culture medium. As the control experiments for NMDA treatment, cortical neurons were incubated with HBSS buffer lacking both NMDA and glycine. Two types of DNMT inhibitor, 5-Aza-2′-deoxycytidine (5AdC) and RG108, were added at 24 h before the treatment. In all experiments, age-matched cultured cortical cells without any treatment were used as the “non-treated group.”

### Immunocytochemistry

Cortical neurons in primary culture were fixed with 4% PFA for 10 min and then washed twice with PBS. The cells were incubated for 10 min at 4°C with 0.2% TritonX-100 containing PBS (PBS-T) for permeabilization. For the staining of 5mC, cells were treated with DNase I, as already mentioned, before being rendered permeable. They were then blocked with 10% goat serum and 1% BSA in PBS-T for 30 min at room temperature. The following primary antibodies, at 4°C, were used: mouse anti-NeuN (cat. no. ab104224, Abcam), mouse anti-GFAP (cat. no. 556327, BD Biosciences), and rabbit monoclonal anti–5mC (cat. no. 28692, Cell Signaling Technology Inc.) antibody. The next day, the cells were incubated with the appropriate secondary antibody, Alexa Fluor 488–labeled goat anti-mouse IgG (cat. no. A11029; Invitrogen) or Alexa Fluor 594–labeled goat anti-rabbit IgG antibodies (cat. no. A11037; Invitrogen). Fluorescence was detected by using an Olympus fluorescence microscope (IX-71; Olympus). Images of random four areas (1.3 mm^2^ each) of each well were taken. Fluorescent images were loaded into the MetaMorph software program (Molecular Devices, Sunnyvale, CA, United States). The number of 5mC-positive cells was counted based on the background fluorescence and the size of nuclei detected by the MetaMorph software program.

### Cell Viability Assay

Cell viability was determined by propidium iodide (PI) staining at 24 h after treatment. Cells were washed twice with PBS and then incubated in 4 μM PI in PBS for 15 min at 37°C. The PI-positive cells were counted in random four areas (1.3 mm^2^ each) of each well and indicated as percentages of the total number of cells, which were stained with Hoechst33342 (cat. no. 346–07951; Dojindo, Kumamoto, Japan).

### Statistical Analysis

All obtained data were analyzed by using the GraphPad Prism software (version 5, GraphPad Software, San Diego, CA, United States) and presented as the means ± standard deviation (SD). To define whether the data were normally distributed, Kolmogorov–Smirnov test was used. The unpaired *t*-test was performed for the comparison between 2 groups. Differences among multiple groups were evaluated by using analysis of variance followed by Tukey test as a *post hoc* test. *P*-values of less than 0.05 were considered to indicate statistical significance.

## Results

### The Number of 5mC–Positive Neurons Increased in the Ischemic Core After Transient MCAO

**FIGURE 1:**
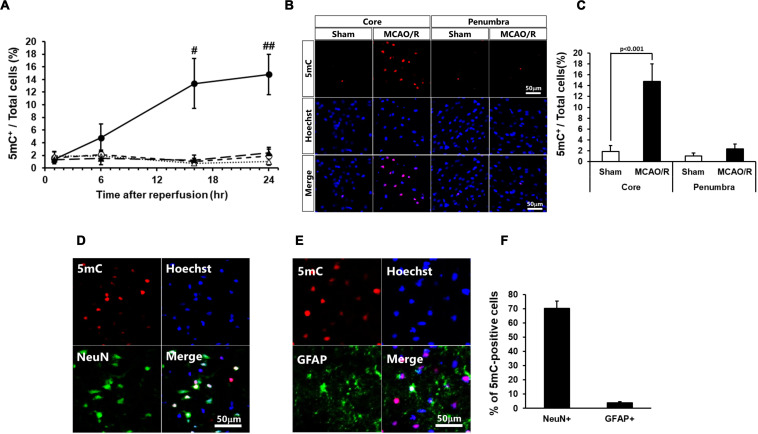
**(A)** Time course of changes in the number of 5mC-positive cells in the core regions (circle) and penumbra regions (triangle) of the sham-operated (open) and ischemic (closed) rats. In these experiments, 6,404–7,845 cells in the core and 2,058–2,404 cells in the penumbra at 1 h, 5,675–8,572 cells in the core and 2,088–2,699 cells in the penumbra at 6 h, 3,699–7,448 cells in the core and 1,896–2,675 cells in the penumbra at 16 h, and 3,585–7,840 cells in the core and 1,919–2,622 cells in the penumbra at 24 h after surgery were counted per animal. Values represent the means ± SD (*n* = 4–7 rats per group). ^#,##^ Significant difference against the sham-operated group of same time point (^#^*p* < 0.01, ^##^*p* < 0.001). **(B,C)** Immunostaining images of 5-methylcytosine (5mC) in the ischemic core and penumbra 24 h after surgery **(B)** and ratio of 5mC-positive cells to total cells (the number of Hoechst-positive cells; **C**). **(D,E)** Double-immunostaining images of 5mC and NeuN **(D)** and 5mC and GFAP **(E)** in the core region of 24 h after surgery. **(F)** Ratio of NeuN- and GFAP-positive cells to 5mC-positive cells in the ischemic core 24 h after surgery. Values represent the means ± SD (*n* = 6 rats per group). The scale bar represents 50 μm.

At first, we examined the time course of changes in the DNA methylation 1, 6, 16, and 24 h after MCAO/R. The number of 5mC-positive cells significantly increased at 16 and 24 h after MCAO/R compared with those of sham-operated rats (Figure 1A). The following studies 24 h after MCAO/R were based on these data. The number of 5mC-positive cells increased in the ischemic core of the cerebral cortex 24 h after MCAO/R compared with that of sham-operated rats (Figures 1B,C). In addition, although there was no statistically significant difference, the number of 5mC-positive cells in the ischemic penumbra of same animals also tended to increase compared with that of sham-operated rats (Figures 1B,C). Next, we performed immunostaining to identify the kind of 5mC-positive cells after transient MCAO. Immunostaining analysis revealed that the 5mC-positive cells in the ischemic core were NeuN-positive neurons (70.2% ± 5.3%), GFAP-positive astrocytes (3.9 ± 0.8%), and others (Figures 1D–F).

### Changes in DNA Methylation After MCAO/R Were Independent of the Levels of DNMT Proteins and mRNAs

**FIGURE 2:**
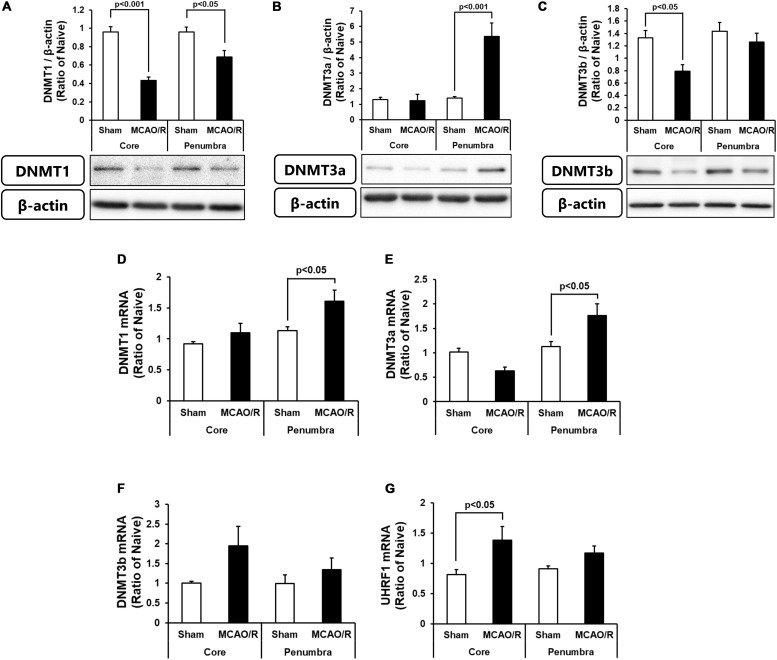
Changes in the protein levels of DNMT1 **(A)**, DNMT3a **(B)**, and DNMT3b **(C)** in the ischemic core and penumbra 24 h after surgery. These DNMTs and β-actin bands were scanned, and the scanned bands were normalized by the naive control on the same blot. β-Actin was used as a loading control. Results are the means ± SD (*n* = 5 rats per group). Changes in the mRNA expression levels of DNMT1 **(D)**, DNMT3a **(E)**, DNMT3b **(F)**, and UHRF1 **(G)** in the ischemic core and penumbra 24 h after surgery. Results are the means ± SD (*n* = 6–7 rats per group).

We next determined the protein and mRNA levels of enzymes for DNA methylation, i.e., DNMT1, DNMT3a, and DNMT3b. As shown in Figure 2A, DNMT1 protein was decreased in both the ischemic core and penumbra, whereas, DNMT1 mRNA was increased in the ischemic penumbra without changes in that in the ischemic core after transient MCAO (Figure 2D). Although there was no change in DNMT3a protein in the ischemic core, that in the ischemic penumbra was significantly increased (Figure 2B). DNMT3a mRNA showed same changes as those of the protein after transient MCAO (Figure 2E). DNMT3b protein was decreased in level in the ischemic core without changes in that in the ischemic penumbra (Figure 2C). DNMT3b mRNA in both the ischemic core and penumbra after transient MCAO was not changed compared with those of the sham-operated group (Figure 2F). Because up-regulation of DNA methylation was not associated with an increase in DNMT levels in the present study, we next examined expression of ubiquitin-like containing PHD ring finger 1 (UHRF1). UHRF1 has the ability to recruit DNMT1 for forming hemimethylated DNA at the replication fork through histone H3 ubiquitination leading to up-regulation of DNA methylation (Liu et al., 2013; Nishiyama et al., 2013). Furthermore, another study revealed that UHRF1 also interacts with DNMT3a and DNMT3b in embryonic stem cells (Meilinger et al., 2009). It was reported that UHRF1 attachment to DNMT1 and DNMT3a is increased by axotomy of primary dorsal root ganglion cells and induces DNA methylation independent of the amounts of enzymes at the promoter of tumor suppressor gene phosphatase and tensin homolog (Oh et al., 2018). As shown in Figure 2G, UHRF1 mRNA expression in the ischemic core was increased, whereas that in the ischemic penumbra was not statistically changed.

### The Number of 5mC-Positive Neurons Increased Immediately After NMDA Treatment

**FIGURE 3:**
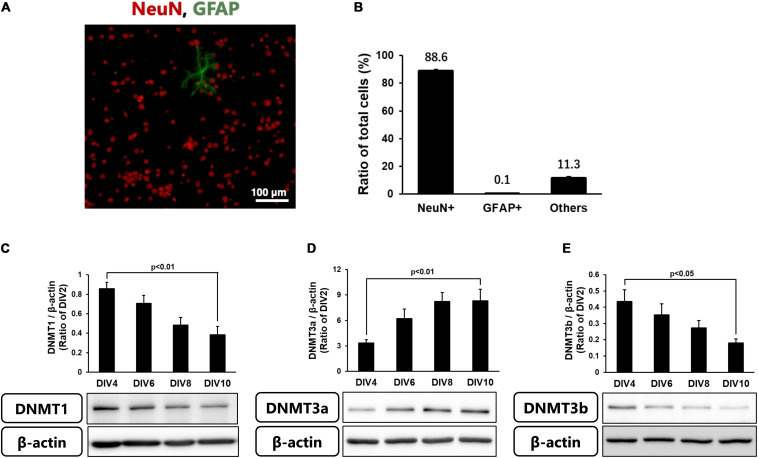
**(A)** Representative images of double-staining for NeuN (Red) and GFAP (green) in cortical neurons in primary culture at 10 DIV. **(B)** Ratio of NeuN- and GFAP-positive cells to total cells (Hoechst-positive cells) at 10 DIV. Images of random four areas (each area of 1.3 mm^2^) of each well were taken, and 1,195–1,320 cells were counted in each well. The scale bar shows 100 μm. Results are the means ± SD (*n* = 4 independent experiments). **(C–E)** Changes in the protein levels of DNMT1 **(C)**, DNMT3a **(D)**, and DNMT3b **(E)** with time in culture. These DNMTs and β-actin bands were scanned, and the scanned bands were normalized by DIV2 cells on the same blot. β-Actin was used as a loading control. Results are the means ± SD (*n* = 5–13 independent experiments).

Because the level of DNA methylation increased in neurons in the ischemic core, we next performed the following experiments using cortical neurons in primary culture. These cells at 10 DIV comprised 88.6% NeuN-positive neurons, a few % of GFAP-positive astrocytes, and other types of cells (Figures 3A,B). We first confirmed the levels of DNMTs in these neurons every 2 days until 10 DIV. Although all DNMTs were expressed in the cultured cortical neurons throughout the experiments, the level of DNMT3a protein was low at 2 DIV and gradually increased until 10 DIV (Figure 3D). On the other hand, DNMT1 and DNMT3b protein levels at 2 DIV were high and then decreased with time in culture (Figures 3C–E).

**FIGURE 4:**
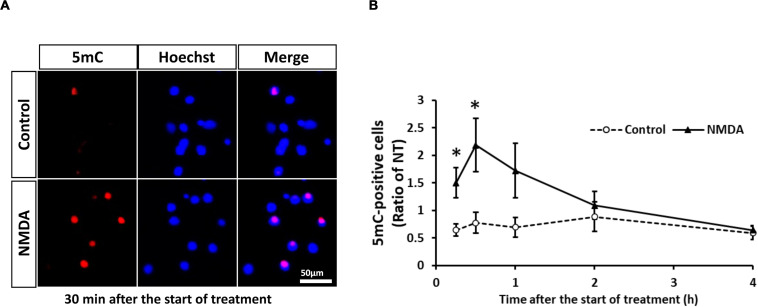
**(A)** Representative images of 5mC immunostaining 30 min after start of treatment. The scale bar shows 50 μm. **(B)** Changes in the ratio of 5mC-positive neurons to total cells (Hoechst-positive cells) after start of NMDA (closed triangle) or vehicle (open circle) treatment. Images of random four areas (each area of 1.3 mm^2^) of each well were taken, and 302–1,250 cells were counted in each well. Results are the means ± SD (*n* = 7 independent experiments). *Significant difference against the control group of the same time point (*p* < 0.05). NT, non-treated group.

**FIGURE 5:**
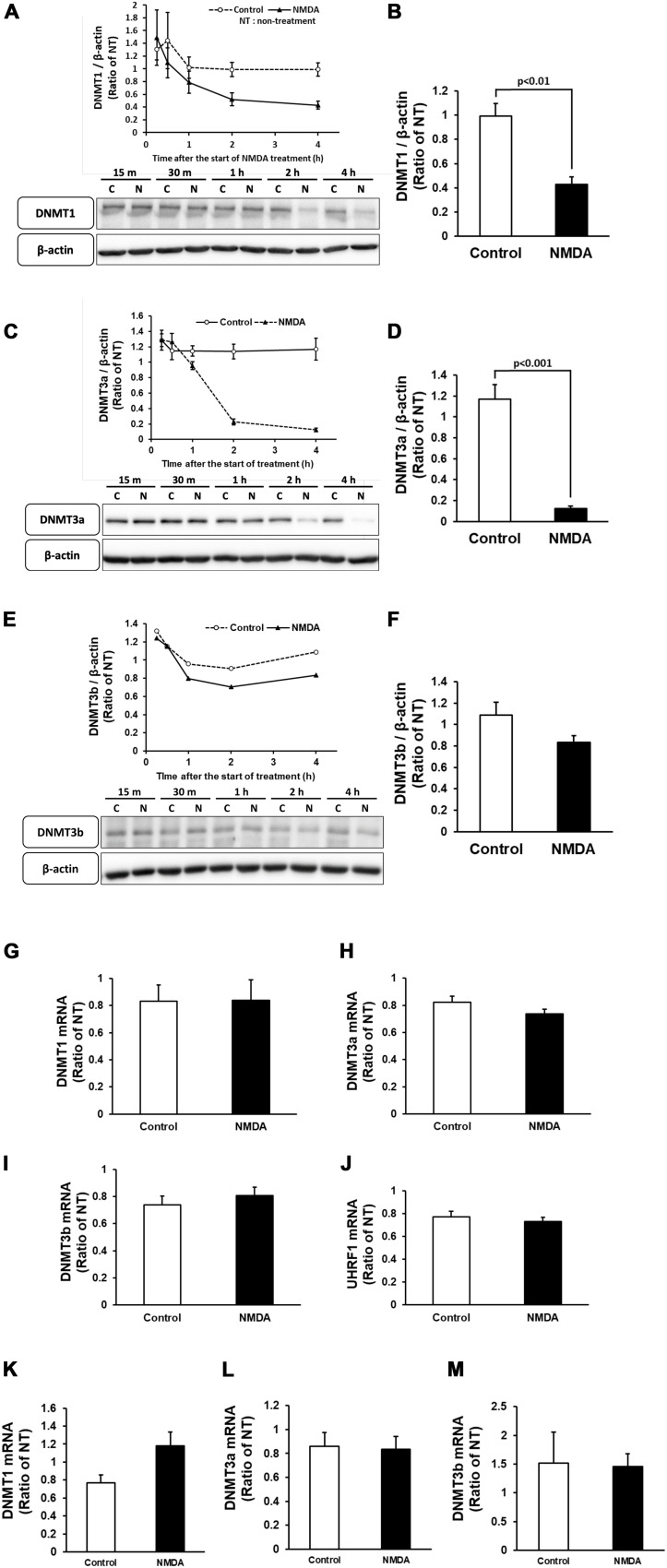
Time course of changes in the protein levels of DNMT1 **(A)**, DNMT3a **(C)**, and DNMT3b **(E)** in cortical neurons in primary culture after NMDA (closed triangle) or vehicle (open circle) treatment. **(B,D,F)** Comparison of the protein levels [DNMT1 **(B)**, DNMT3a **(D)**, and DNMT3b **(F)**] in NMDA-treated group and vehicle-treated control group at 4 h of treatment. These DNMTs and β-actin bands were scanned, and the scanned bands were normalized by the non-treated control on the same blot. β-Actin was used as a loading control. Results are the means ± SD (*n* = 4–5 independent experiments). **(G–J)** Changes in the mRNA expression levels of DNMT1 **(G)**, DNMT3a **(H)**, DNMT3b **(I)**, and UHRF1 **(J)** at 30 min of treatment. Results are the means ± SD (*n* = 4 independent experiments). **(K–M)** Changes in the mRNA expression levels of DNMT1 **(K)**, DNMT3a **(L)**, and DNMT3b **(M)** at 4 h of treatment. Results are the means ± SD (*n* = 5 independent experiments). NT, non-treated group; C, control group; N, NMDA-treated group. 15 m, 15 min, 30 m, 30 min.

Neuronal cell injury was induced by NMDA treatment at 10 DIV, as previously reported (Yamada et al., 2019). We next determined the number of 5mC-positive neurons after NMDA treatment. The number of 5mC-positive neurons markedly increased 30 min after the start of NMDA treatment and then decreased to the same level as that of the control group (Figures 4A,B). Western blotting analysis revealed that the expression of all DNMTs remained at the control levels until 1 h after NMDA treatment, when a pronounced number of 5mC-positive neurons were detected (Figures 5A,C,E). Then these protein levels tended to decrease from 2 to 4 h after the start of NMDA treatment. We next focused on the events at 4 h after NMDA treatment when DNA methylation returned to the level of control in neurons. The DNMT3a protein level showed a 0.11-fold decrease compared with the control group at that time point (Figure 5D). DNMT1 and DNMT3b proteins were 0.43- and 0.77-fold, respectively (Figures 5B,F). We detected no significant changes in the mRNA levels of those enzymes at 30 min and 4 h after the start of NMDA treatment compared with those of the vehicle-treated groups (Figures 5G–I,K–M). We also investigated the level of UHRF1 mRNA as in the *in vivo* study. UHRF1 mRNA expression was not changed at 30 min after the start of NMDA treatment, when the number of 5mC-positive neurons had increased (Figure 5J).

### DNMT Inhibitor RG108 Had a Protective Effect Against NMDA-Induced Neuronal Cell Death

**FIGURE 6:**
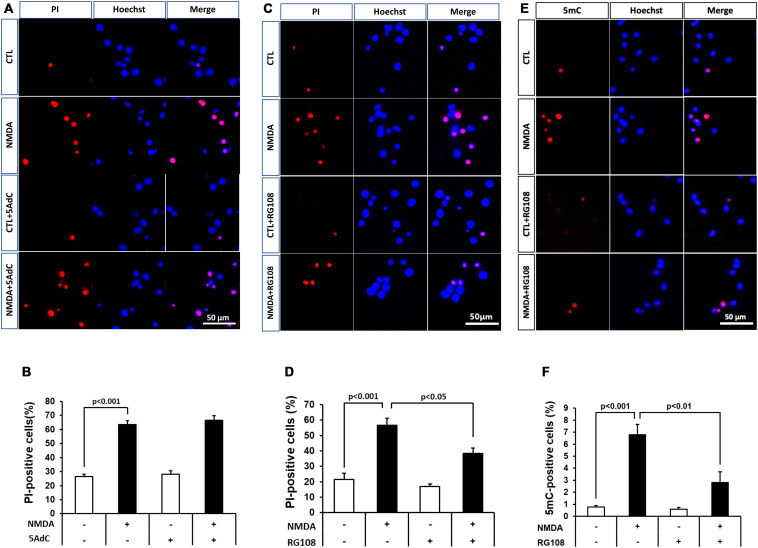
**(A,C)** Effects of DNMT inhibitors 5AdC (10 μM; **A**) and RG108 (100 μM; **C**) on NMDA-induced cell injury. Representative images of PI staining are shown. The scale bar indicates 50 μm. **(B,D)** PI-positive cells were counted as dead cells and are shown as the ratio to total cells (Hoechst-positive cells). Counted cell number was 933–1,697 **(B)** and 736–1,087 **(D)** in each well. **(E,F)** Effects of DNMT inhibitor RG108 (100 μM) on NMDA-induced DNA methylation 30 min after start of treatment. **(E)** 5mC-positive cells were counted and are shown as the ratio to total cells (Hoechst-positive cells). Counted cell number was 731–1,049 **(F)** in each well. The scale bar shows 50 μm. Images of random four areas (each area of 1.3 mm^2^) of each well were taken. Results are the means ± SD (*n* = 4–5 independent experiments). PI, propidium iodide; CTL, control group.

**FIGURE 7:**
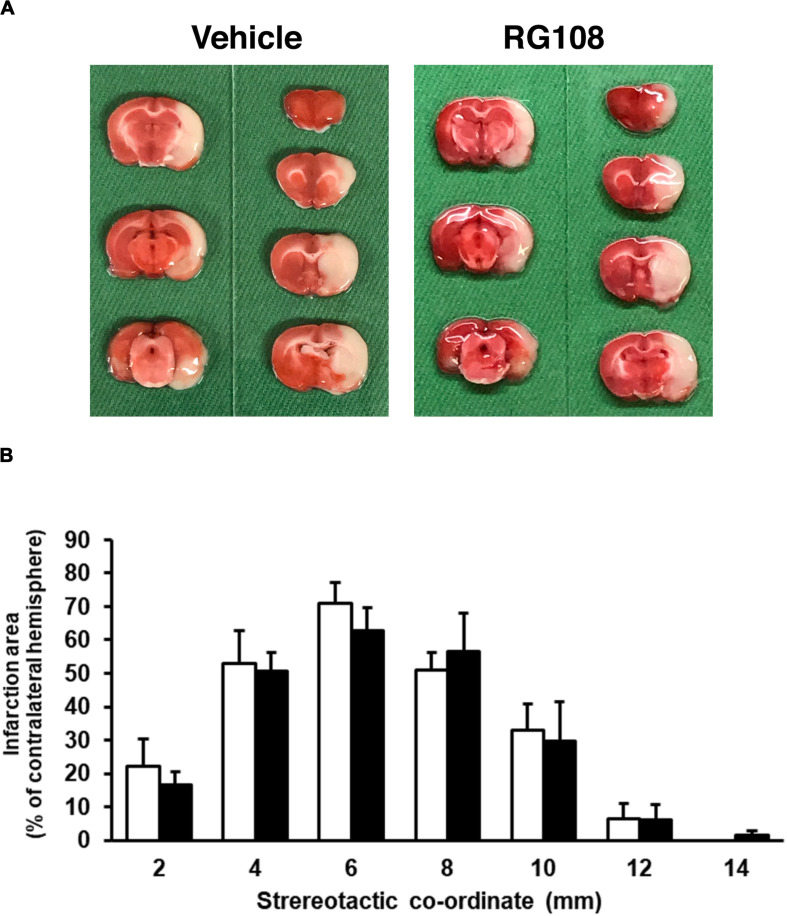
**(A)** Representative photographs of TTC staining from vehicle- and RG108-treated MCAO/R rats. **(B)** The infarct area of hemisphere at 2, 4, 6, 8, 10, 12, and 14 mm from the forebrain in the vehicle-treated (white bars) and RG108-treated (black bars) MCAO/R rats 24 h after surgery. The infarct areas are expressed as percentages of contralateral hemisphere. Values represent the mean ± SD (*n* = 4 each).

Because DNA methylation was increased in neurons in the pathological condition, we further examined the effect of DNMT inhibitors on NMDA-induced neuronal injury. Nucleoside analogs 5AdC and RG108, which are known as non-nucleoside DNMT inhibitors, were used in the present study. We demonstrated that the number of PI-positive cells with NMDA treatment was not influenced by the 5AdC treatment (Figures 6A,B). On the other hand, the increased number of PI-positive cells caused by NMDA-induced neurotoxicity was decreased by treatment with RG108 (Figures 6C,D). Finally, we examined the effect of RG108 on the number of 5mC-positive cells after NMDA treatment to verify a possible relationship between NMDA-induced neuronal injury and DNA methylation in neurons. The increased number of 5mC-positive cells induced by NMDA treatment was inhibited by treatment with RG108 (Figures 6E,F). Furthermore, we tested the effect of DNMT inhibitor on infarct size after transient MCAO. There was no statistically significant effect on infarct areas (Figure 7).

## Discussion

Our results showed that aberrant DNA methylation, which was accompanied by neuronal cell death, occurred in the early stage of ischemic injury. Therefore, the regulation of gene expression that was related to neuronal cell death might be induced by this epigenetic modification in the ischemic brain. Up-regulation of DNA methylation was not correlated with the levels of DNMT proteins and mRNAs in the present study. Thus, changes in the activity of DNMTs might be more important determinants of the enhanced DNA methylation in neurons under ischemic conditions. Because it has been reported that some epigenetic regulators such as UHRF1 are associated with DNA methylation, we examined UHRF1 mRNA as one such candidate. Although the level of UHRF1 mRNA was accompanied by DNA methylation *in vivo*, there was no change in the level of that in injured neurons with up-regulated DNA methylation. UHRF1 is highly expressed in cancer cells, and it is thought that its level is correlated with the ability of cancer cells to proliferate (Ashraf et al., 2017). As accumulating evidences indicated that UHRF1 is also related to the S phase entry in normal cells (Bonapace et al., 2002; Blanchart et al., 2018), UHRF1 has been thought to play a role in cell proliferation. Therefore, up-regulation of UHRF1 mRNA seen in our *in vivo* results might be related to the level of DNA methylation in dividing cells such as glial and endothelial cells in the brain under ischemic conditions. In the present study, we performed immunostaining to determine the level of DNA methylation. As whole-genome DNA methylation is detected by immunostaining, it has been considered to be difficult to measure the status of DNA methylation of specific DNA loci (Kurdyukov and Bullock, 2016). Therefore, there is a possibility that local DNA methylation, which cannot be determined by immunostaining, was enhanced in non-neuronal cells, including endothelial cells, and that UHRF1 may have contributed to these changes. In this sense, it was reported that DNA methylation influences the expression of the angiostatic factor thrombospondin 1 in cerebral endothelial cells during oxygen-glucose deprivation (Hu et al., 2006). These reports suggested that angiogenesis regulated by DNA methylation in the endothelial cells may also contribute to the pathogenesis of stroke. On the other hand, even though DNA methylation tended to increase in level in the ischemic penumbra, the expression of DNMT3a mRNA and protein was significantly increased in this region. Although it was reported that DNA methylation is increased in the ischemic penumbra 24 h after MCAO/R (Morris-Blanco et al., 2019), some other study showed that the methylation increased at the late stages of photoreceptor cell death (Farinelli et al., 2014). Therefore, we will need to examine cellular sources and the time course of changes in DNA methylation in detail to elucidate the pathological alteration in stroke.

As we previously reported that NMDA receptor subunits GluN1, GluN2A, and GluN2B show increased expression with time in cortical neurons in primary culture (Yamada et al., 2018), we examined changes in DNA methylation after NMDA-induced neuronal injury, which mimics cerebral ischemia-induced neuronal cell injury *in vivo*. The amounts of DNMT proteins were changed with stages of development (Feng et al., 2005; Watanabe et al., 2006). In addition, it was reported that DNMT1 and DNMT3a were expressed differentially during brain and spinal cord maturation in mice and that DNMT3a, but not other DNMTs, had the ability to promote cell death of motor neurons (Chestnut et al., 2011). In this sense, although DNMT1, 3a, and 3b proteins were expressed until the day of treatment, DNMT3a was increased with stages of development in cultured neurons in the present study. It might be a possible involvement of DNMT3a in NMDA toxicity in neurons, although we could not fully demonstrate the most important DNMTs. DNA methylation increased immediately after the start of NMDA treatment and then returned to the level of the control group. When DNA methylation level returned to the control level, DNMT3a protein was the most decreased one among all of the DNMTs. Especially as a *de novo* DNA methyltransferase, DNMT3b has been shown to play a key role in neurogenesis in the embryonic stage, whereas, DNMT3a contributes to DNA methylation in both embryogenesis and postnatal development in the brain (Feng et al., 2005; Watanabe et al., 2006). Our results also showed that only the expression of DNMT3a protein was increased with time in culture compared with other DNMTs. Considering that DNMT3a was the most abundant of the DNMTs in mature cortical neurons, DNMT3a might be needed to maintain the increased level of DNA methylation elicited by NMDA treatment. Furthermore, DNA methylation was transient in our *in vitro* experiments, suggesting that DNA methylation could be forcibly suppressed in cells to induce DNA demethylation. 5-Hydroxymethyl cytosine (5hmC) is derived from 5mC as an enzymatic intermediate of DNA demethylation, and it was reported that 5hmC is more abundant in the brain than in other tissues in adult mice (Globisch et al., 2010). Another study reported that 5hmC-positive cells co-localize with mature neuron markers in the cerebellum and hippocampus and that these double-positive cells increased in number with neuronal maturation (Szulwach et al., 2011). Moreover, the regulation of gene expression by DNA demethylation is related to neuronal synaptic function and/or intrinsic membrane activity (Meadows et al., 2015, 2016). These findings suggest that DNA demethylation might be important for neuronal function. Recently, it was reported that DNA demethylation is increased in the ischemic penumbra after MCAO/R and that this increase might be potentially linked to a decrease in infarct volume (Morris-Blanco et al., 2019). Based on these results, DNA demethylation after cerebral ischemia may be a compensatory response against ischemic injury. Conversely, DNA methylation seems to be an initiation factor of neuronal cell death, and is consistent with our results. The neuroprotective effect of RG108 against NMDA-induced cell injury supports this hypothesis. In this way, it is possible that there is a close relationship between the regulation of gene expression by DNA methylation and neuronal cell death under the ischemic condition. Therefore, we will need to determine its target gene to know more about DNA methylation after cerebral ischemia in a future study. There were no changes in the levels of DNMTs mRNA when the levels of protein were decreased 4 h after NMDA treatment. In particular, DNMT3a has been reported to be degraded by the ubiquitin proteasome system in cancer cells (Jia et al., 2016). Similar to this report, our results indicated that DNMT protein level might be regulated through posttranslational degradation.

In the present study, the NMDA-induced neurotoxicity was attenuated by RG108 treatment that was accompanied by inhibition of DNA methylation. This result suggested a possible relationship between cell injury induced by NMDA neurotoxicity and DNA methylation. However, RG108 could not reduce the cell death completely in the present study. RG108 is known as a non-nucleoside DNMT inhibitor, which is different from other nucleoside inhibitors such as 5AdC. It was reported that a non-nucleoside inhibitor has a lower inhibition effect compared with other nucleoside inhibitors (Rondelet et al., 2017). This would be one of the reasons why the protective effect of RG108 was not adequate to completely halt cell death in this study. Whereas RG108 is capable of associating with catalytic domains of DNMTs directly, 5AdC needs to be incorporated into the DNA during the S phase of the cell cycle in order to work (Gnyszka et al., 2013). Therefore, 5AdC might have failed to inhibit the activity of DNMT in non-dividing neurons, indicating no protective effect of it on NMDA-induced cell injury.

Although, in the present study, we have investigated the effect of RG108 on the infarct size after MCAO/R, the infarct area was not affected, regardless of administration with RG108. It was reported that DNMT inhibitor could reduce infarct volume in mice subjected to 30 min MCAO (Endres et al., 2000) and in rats that underwent a 45 min MCAO (Dock et al., 2015). In addition, DNA methylation was increased in rats subjected to 30 min MCAO but not 2 h occlusion (Dock et al., 2015). As pathological alterations after cerebral ischemia are very complex, it is possible that the relationship between DNA methylation and cell injury after cerebral ischemia may depend on the severity of cerebral ischemic condition, the time course, brain regions, and the different cell types. In addition, there were some limitations in the present study that double-staining for PI- and 5mC-positive cells has not been addressed in cultured neurons. It remains to be clarified whether 5mC-positive cells in control and NMDA-treated groups are injured. Furthermore, site-specific DNA modification would correlate with cell survival or death. Therefore, we will need to test in several *in vitro* and *in vivo* disease conditions and at multiple time points and to carefully dissect the relationship between DNA methylation and cell damages.

In the present study, we demonstrated that DNA methylation increased in neurons after ischemic injury, and this might lead to cell death. We also showed that this DNA methylation was not dependent on the levels of the proteins and mRNAs of DNA methylation enzymes and preceded neuronal cell death. Preventing this epigenetic modification and subsequent changes in related-gene expression of neurons might become a valuable therapeutic strategy for stroke.

## Data Availability Statement

All datasets presented in this study are included in the article/supplementary material.

## Ethics Statement

The animal study was reviewed and approved by the Committee of Animal Care and Welfare of Tokyo University of Pharmacy and Life Sciences.

## Author Contributions

MA, HH, and NT designed the study. MA, KM, KK, and RK performed the experiments. HH, BY, and NT contributed analytical tools and discussed the results. MA and NT wrote the manuscript. All authors read and approved the final manuscript.

## Conflict of Interest

The authors declare that the research was conducted in the absence of any commercial or financial relationships that could be construed as a potential conflict of interest.
